# A randomised trial of the bone formation after maxillary sinus floor augmentation with bovine hydroxyapatite (Cerabone®) and Photobiomodulation: histomorphometric and immunohistochemical analysis

**DOI:** 10.4317/jced.60594

**Published:** 2023-07-01

**Authors:** Fabio-André Klassmann, Edilson Ervolino, Leandro-Eduardo Kluppel, Letícia-Helena Theodoro, Gabriel Mulinari-Santos, Valdir-Gouveia Garcia

**Affiliations:** 1 Latin American Institute of Dental Research and Teaching, School of Dentistry Ilapeo, Curitiba, Paraná, Brazil; 2 Department of Basic Sciences, School of Dentistry, São Paulo State University (UNESP), Araçatuba, São Paulo, Brazil; 3 Department of Diagnosis and Surgery, School of Dentistry, São Paulo State University (UNESP), Araçatuba, São Paulo, Brazil

## Abstract

**Background:**

The use of non-autogenous biomaterial to increase bone height in the maxillary sinus has been shown to be effective, but the results are still inconclusive.

**Material and Methods:**

Eight participants were selected and included in the research. After surgical access with osteotomy on the lateral wall of both maxillary sinuses, these were filled with Cerabone®. Then, by blind randomization, they received one of the following treatments: Filling with Cerabone® (Control group); treatment with Photobiomodulation (PBM), filling with Cerabone® and treatment with low-power laser (PBM group). Biopsies were obtained 30 days after the surgery, using a 2.8 mm internal diameter trephine bur. Qualitative and quantitative histological analyzes were performed and immunohistochemical analyzes of osteocalcin (OCN) and tartrate-resistant acid phosphatase (TRAP) were performed with scores for each of the biological events.

**Results:**

The Cerabone® biomaterial demonstrated a high degree of biocompatibility. New bone formation was observed in both groups. In the PBM group, there was greater bone formation and newly formed tissue in an advanced state of bone maturation. The immunostaining of OCN was greater at 30 days in the PBM group than in the control. There was no significant difference in TRAP immunostaining at 30 days between the groups.

**Conclusions:**

Low-power laser-mediated by PBM promoted greater bone formation; the newly formed tissue showed a more advanced state of bone maturation in maxillary sinuses filled with Cerabone® biomaterial and treatment with PBM, within the 30-day evaluation period.

** Key words:**Sinus floor augmentation, dental implants, bone and bones, low-level light therapy.

## Introduction

Tooth loss in the posterior maxilla can result in a clinical challenge for rehabilitation with oral implants (1). Mainly when the alveolar bone wall of the maxillary sinus reveals low quality and quantity of bone due to maxillary sinus pneumatization (1-3). The insufficient residual bone height may compromise the primary implant stability and osseointegration (3). In these conditions, treatments without sinus floor augmentation such as using zygomatic implants or short implants (1) arise, but they must be used cautiously.

In the search for a predicTable technique of sinus floor augmentation, not only procedures for accessing the maxillary sinus have been described (2-5) as well as the use or not biomaterials in this area (6-7). The use of biomaterials for sinus floor augmentation can present limitations, including not enough bone formation after a long healing period (4-7). Combinations of biomaterials with platelet concentrate have been proposed to enhance and accelerate bone regeneration, although these techniques are relatively complex and discussed (8). Therefore, reliable solutions for sinus floor augmentation are still being investigated.

Autogenous bone is considered the gold standard among biomaterials since its osteoinductive and osteogenic properties and no undesirable immune responses (9,10). However, its use has limitations, principally when there is a need for a large bone volume or when the patient does not have donor areas with a large bone amount (9,10). Likewise, this procedure requires a second surgical area, which is disagreeable for patients, in addition to risks of nerve injury, trismus, mandibular fracture, pulp morbidity, changes in sensitivity, and damage to vascularization (11).

Alternatives have been proposed, accentuating the use of homogeneous, xenogeneic, and alloplastic biomaterials (12,13). The use of xenogeneic biomaterial is a safety option, since it is biocompatible, easy to obtain, and low cost, besides being obtained in larger volumes. The bone of bovine origin is the most used, but the use of porcine and equine bone also demonstrated osteoconductive properties and small osteoinductive properties (13). More recently, a new xenogeneic biomaterial has been highlighted, Cerabone® (© Institut Straumann AG). It is a biomaterial from bovine spongy bone composed of calcium phosphate with 100% pure hydroxyapatite, porous of 65-80%, with an average pore size between 600 and 900 µm. The number of research evaluating its use is recent and limited, mainly in sinus floor augmentation (14,15) which requires the development of new research.

On the other hand, there has been a growing interest in the use of photonic therapy in dentistry, particularly the use of low-power laser (LPL) or light-emitting diode which can promote the acceleration of wound healing, reduce pain and inflammation, conceptually termed photobiomodulation (PBM) (16). The benefits of PBM in bone healing have been demonstrated in different conditions and experimental models (17-21), including sinus floor augmentation (22,23). Results of these studies revealed that the sinus floor augmentation with biomaterial promoted a significantly higher bone formed and a less residual amount of biomaterial in combination with laser therapy (22). In addition, the use of LPL with the simultaneous biomaterial and implant placement promoted faster bone healing and higher bone density in the treated areas, indicating a consistent treatment for bone regeneration (22-24).

Thus, this study aims to evaluate bone healing in maxillary sinuses filled with Cerabone®, treated or not with PBM mediated with LPL. The hypothesis of the present study is that PBM can accelerate and enhance bone formation in sinus floor augmentation with Cerabone®, while the null hypothesis is that PBM does not contribute to this condition.

## Material and Methods

-Study Populations and Ethical Statement

All participants of the present study were selected in a single institution, in the clinics of Ilapeo College (Curitiba, PR, Brazil). The study protocol was approved by Brazil Platform (#4.412.714, 29 April 2020), the Brazilian Federal Government body for the Control of Clinical Research in Human Beings. The research participants, after receiving all the research information and clarifying all their doubts, signed a Free and Informed Consent Form (FICF). The structure of this study followed the guidelines of the CONSORT checklist.

-Inclusion and Exclusion Criteria

The following inclusion criteria were followed: (i) participants with the indication of installation of dental implants in the posterior region of the maxilla; (ii) need for bone reconstruction in the maxillary sinus (sinus lift), bilaterally; (iii) residual bone remnant between the alveolar ridge and the floor of the maxillary sinus between 5 and 7 mm; (iv) age over 18 years; (v) women who are not in the gestational period. The following exclusion criteria were adopted: (i) patients with diabetes (controlled or not); (ii) patients with systemic disorders and/or who use drugs that interfere with bone metabolism; (iii) history of cancer, treated with irradiation or chemotherapy; (iv) history of alcohol abuse; (v) smokers; (vi) non-FICF subscribers.

-Sample Size 

The calculation of the larger study sample was performed for two groups with a single evaluation period and resulted in a minimum sample size, considering a test power of 85% and a significance level equal to 0.05 (5%), equivalent to 12 participants per group. However, in this study, we present the results of 8 participants, which is equivalent to more than 50% of the sample size, since problems contrary to our will prevented the inclusion of a larger number of participants, due to the SARS-COV2 pandemic.

-Study Design

This is a split-mouth triple-blind study where the research participants, the surgeon, and the histomorphological and immunohistochemical evaluator had no prior knowledge about the treatment. All study participants underwent blinding and sinus/procedure randomization for the application of PBM, distinguishing two groups: The control group, where the maxillary sinuses filled with the biomaterial did not receive laser treatment, and the PBM group (test), where the maxillary sinus, after being filled with the biomaterial, were treated with low-level laser therapy.

-Blinding and randomization

Blinding and randomization of participants were performed as follows: each participant included in the study was randomly assigned an identification number. Next, a random allocation to the side to be applied the laser (PBM) was carried out, which was as follows: eight sealed envelopes without identification contained a single note written ¨left side¨ or ¨right side¨. At the time of surgery, each patient chose one. When choosing the envelope, it was defined on which side of each patient the PBM therapy was applied.

-Clinical procedures

All study participants included in the research underwent the surgical procedure of lifting the maxillary sinus, bilaterally, with the lateral bone window technique (5).

**Figure 1:**
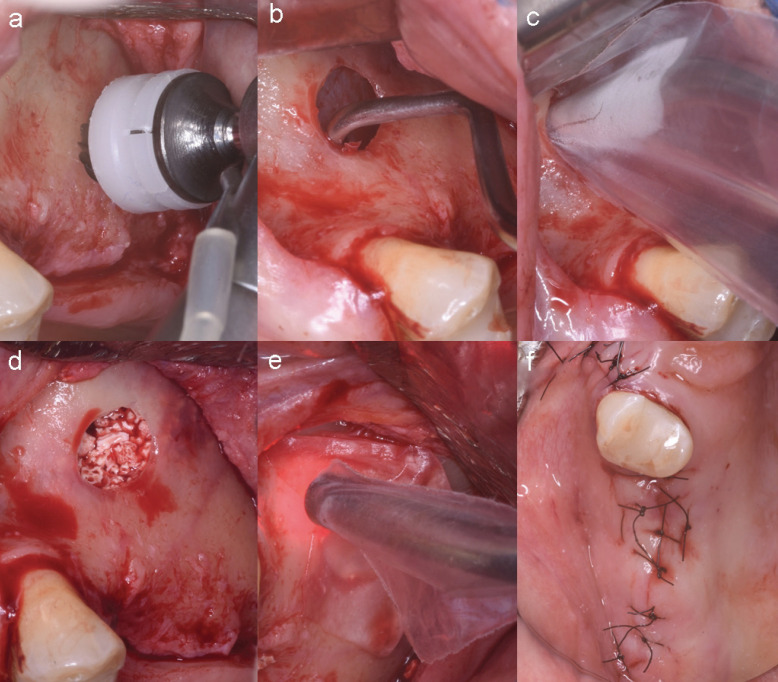
Surgical protocol and treatment (a) Bone wall of the maxillary sinus exposed and osteotomy drill in position to obtain the bone window of the maxillary sinus; (b) Bone window of the maxillary sinus performed and detachment of Schneider’s membrane; (c) Transsurgical application of photobiomodulation, (d) Biomaterial filling the maxillary sinus; (e) Membrane (Jason membrane®) closing the window and trans surgical application of photobiomodulation in a second moment; (f) Flap stabilization with sutures.

Before starting the procedure, all participants rinsed the oral cavity with 0.12% chlorhexidine for one minute. After anaesthesia with 2% Mepivacaine (DFL, São Paulo, SP, Brazil) a horizontal mucosal incision was made along the bone in the edentulous area. Then, two relaxing vertical incisions were made and a mucoperiosteal flap was raised. After flap elevation, the site of access to the maxillary sinus was determined on the anterior bone wall of the maxillary sinus. The osteotomy was performed with was performed with a drill (Polymers 6.5 WF Cirúrgicos, São Paulo, SP, Brazil). All cortical bone was removed in approximately 10 mm using a surgical motor at 700 rpm under abundant irrigation with saline solution until exposing the sinus membrane, which was carefully detached so as not to cause perforation of the sinus membrane (Fig. 1a-c).

-Biomaterial Graft

Immediately after opening the lateral bone window and detaching the sinus membrane, trans-surgical application of PBM with LPL was performed on the selected side. Then, the maxillary sinuses, both on the laser-treated side and on the control side without laser treatment, received a Cerabone® biomaterial graft (Straumann Brasil, Curitiba, PR, Brazil) with granulation of 1-2 mm and volume of 1cc. After placement of the biomaterial, the lateral bone window of the maxillary sinus was obliterated with a resorbable Jason membrane® (Straumann Brasil, Curitiba, PR, Brazil). Next, the flap was repositioned without tension and stabilized with sutures (Monovrycril 4-0, Shalon Medical, Goiânia, GO, Brazil) (Fig. 1d-f).

-Postoperative Guidelines

Written and verbal postoperative recommendations were presented to the participants, as well as the following medication was prescribed: Amoxicillin (500mg, 8/8 hour, 7 days), Celebra (200mg, 12/12 hour, 3 days, Dipyrone (500mg, 6/6 hour, 3 days) and rinse with 0.12% chlorhexidine starting 24 hours after surgery for seven days.

-Laser Treatment

The Laser Dual Therapy XT (DMC Equipamentos, São Carlos, São Paulo, Brazil) was used, which has the following technical characteristics: Diode laser, GaAlAs, 660 nm±10nm (Visible, red), and 808 nm±10nm (Infrared), 100 mW ±20%, optical fiber diameter of 600 µm. The laser was used in two moments: in the trans-surgical after displacement of the sinus membrane and after filling the maxillary sinus with the biomaterial, following the application protocol: First, it was 660nm, 0,1W, 20 seconds per point in 5 points with the total energy of 10J, punctual contact, and continuous emission application mode. The second moment was during the postoperative period, where the laser was applied for 15 days, with intervals of 48 hours between sessions with the following protocol: 808nm, 0.1W, 20 seconds per point, total energy of 10J, application mode punctual contact, and continuous emission. 

-Sample collection

**Figure 2:**
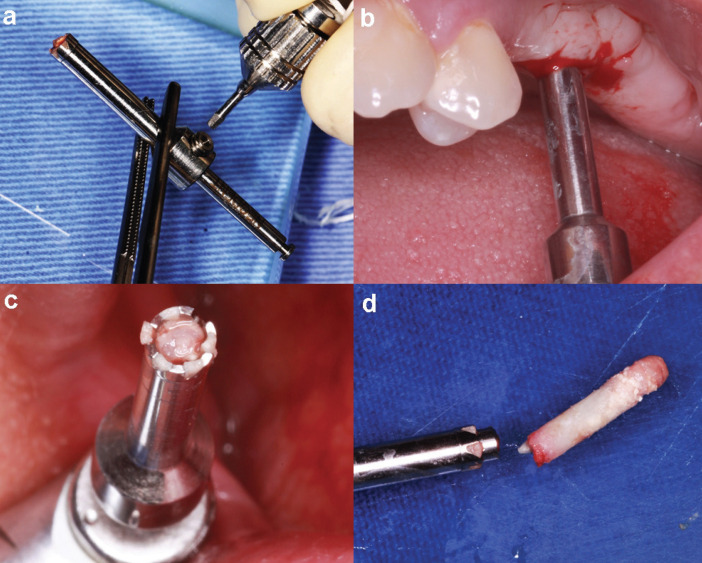
Obtaining bone biopsies. (a) Installation of a trephine bur penetration depth limiter; (b) Trephine bur in position for tissue removal; (c) View of the biological material obtained inside the trephine bur; (d) Bone biopsy obtained.

Thirty days after the surgery, the collected bone biopsy was performed. After the area was anaesthetized, a trephine drill with an internal diameter of 2.8 mm and an external diameter of 3.8 mm (Neodent, Curitiba, PR, Brazil) was used with a rotation of 700 rpm coupled to an implant motor. A depth stop was fitted to the body of this trephine drill, so all biopsies had the same length. The collection region was previously planned to be the area between the region where the future implants will be installed, with the trephine drill parallel to the sites of these implants, not interfering with their subsequent installation (Fig. 2).

-Laboratory and analysis procedures

-Histologic procedures

After 48 hours in 10% formalin, the biopsies underwent the laboratory for demineralization in a demineralizing solution (PBS plus 10% ethylene diamine tetra acetic acid – EDTA for 60 days). After it was washed in running water, dehydrated, cleared, impregnated, embedded in paraffin and sectioned in a microtome with 4 µm thick. Semi-serial sections were performed and captured on histological slides.

For the histopathological analysis of the tissues and histometric analysis of the percentage of bone (PB), the percentage of soft tissues (PS), and the percentage residual of the bone graft biomaterial (PBGM), the histological sections were submitted to staining with hematoxylin-eosin (HE). In other slides, the sessions were treated using the immunohistochemical technique to detect osteocalcin (OCN) and tartrate resistant acid phosphatase (TRAP). Sample analyses were performed by an expert examiner (EE), who was blinded to the samples.

-Histomorphological analysis of the samples

**Figure 3:**
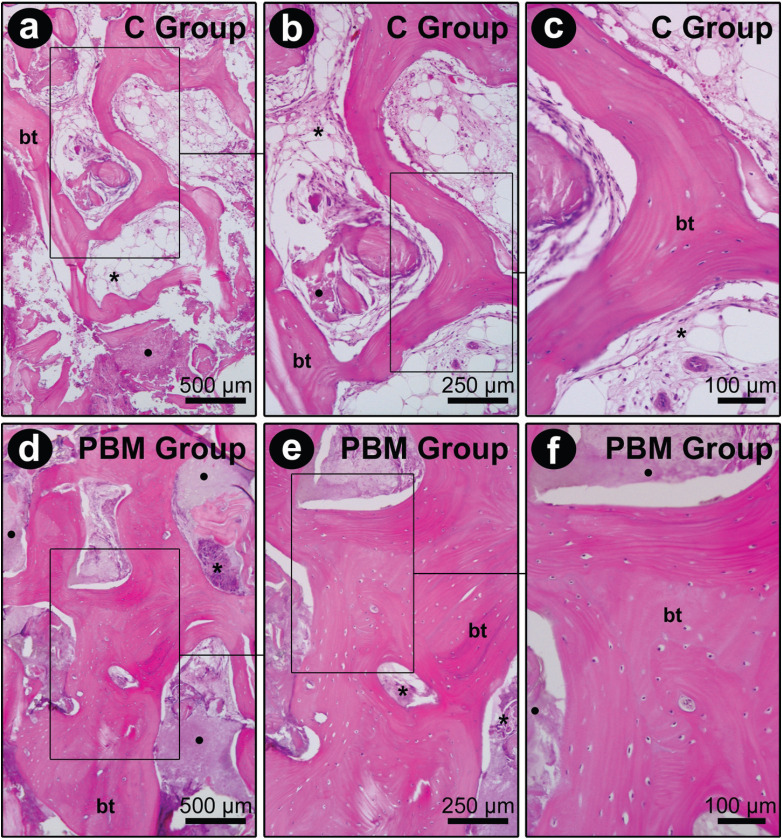
Histological aspect of samples from the surgical site of C (a–c) and PBM groups (d–f). Photomicrographs show a large amount of bone tissue in the PBM group compared to the C group. Abbreviations and symbols: bt, bone tissue; *, soft tissue; •, fragments of the biomaterial. Staining: Hematoxylin and eosin. Original magnifications: a, d, 50x; b, e, 100x; c, f, 200x. Scale bars: a, d, 500 µm; b, e, 250 µm; c, f, 100 µm.

**Figure 4:**
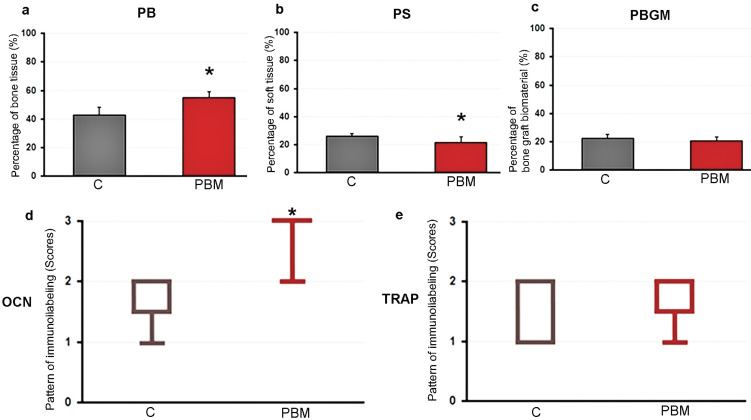
Graphs showing the histomorphometric analysis of PB (a), PS (b), PBGM (c) and the immunostaining pattern for OCN (d) TRAP (e) at the surgical site in the C and PBM groups. Mann-Whitney U test, a non parametric test. Symbol: *, a statistically significant difference compared to group C.

Histological sections were analyzed under bright field illumination in an optical microscope (Axiolab, Carl Zeiss). Two histological sections (50 μm apart) from the center of each sample were used to perform the histometric analysis. In each section, two areas of 5.88 mm2 (2.8 mm x 2.1 mm) were analyzed with a 50x magnification, one located in the most rostral portion and the other in the intermediate portion of the sample. The PB, PS, and PBGM were considered in each sample (Fig. 3). The PS consisted of the area occupied by connective tissue, adipose tissue, and hematopoietic tissue. Images of the areas of interest described above were captured using a digital camera (AxioCam, Carl Zeiss MicroImaging GmbH, 07740 Jena, Germany) coupled to an optical microscope (Axiolab, Carl Zeiss MicroImaging GmbH, 07740 Jena, Germany) connected to a microcomputer. With the aid of the image analysis program (Axiovision 4.8.2, Carl Zeiss MicroImaging GmbH, 07740 Jena, Germany) PB, PS, and PBGM were measured (Fig. 4a-c).

-Immunohistochemical procedures

For immunohistochemical analyses, histological sections were deparaffinized with xylol and hydrated in a decreasing series of ethanol. Antigenic retrieval was performed by immersing the histological slides in 10 mM citrate buffer and pH 6.0 (Spring Bioscience), in a pressurized chamber (Decloaking Chamber®, Biocare Medical) at 95°C. At the end of each stage of the immunohistochemical reaction, the histological slides were washed in 0.1 M PBS and pH 7.4.

Subsequently, the slides were immersed in a solution consisting of 3% hydrogen peroxide, for 1 hour, and in a solution consisting of 4% skimmed milk powder, also for 1 hour, to block peroxidase and endogenous biotin, respectively. Blocking of nonspecific sites was performed in a solution consisting of 1.5% bovine serum albumin in PBS plus 0.05% Triton® X-100 (Sigma-Aldrich) for 12 hours.

Slides containing samples from each experimental group were divided into 2 batches. Each batch was incubated for 24 hours with one of the following primary antibodies: anti-OCN (Abcam Laboratories) and anti-TRAP (Santa Cruz Laboratories). Then, the sections were incubated with biotinylated secondary antibody (Vector Laboratories) for 2 hours and subsequently treated with streptavidin conjugated to horseradish peroxidase (Vector Laboratories) for 2 hours. Development was performed using the compound 3,3’-diaminobenzidine (Vector Laboratories) as a chromogen. The specimens were counterstained with Harris Hematoxylin, then dehydrated in ethanol, cleared in xylol, and covered with mounting medium and glass coverslips. As a negative control, the specimens were submitted to the same procedures previously described, suppressing only the primary antibody use. All stages of the immunohistochemical reaction were based on the previously described protocol (25).

-Immunohistochemical analysis of samples

For OCN, semi-quantitative analysis was performed applying evaluation scores of immunoreactive cells (IR) which were: SCORE 0: null immunostaining means total absence of IR cells and absence of extracellular matrix (ECM) marking; SCORE 1: Low immunostaining pattern (1/4 of IR cells and weak staining on ECM); SCORE 2: Moderate pattern of immunostaining (1/2 of IR cells and moderate staining on ECM); SCORE 3: High pattern of immunostaining (3/4 of cells IR and moderate labeling on the ECM).

For TRAP analysis, semi-quantitative analysis was carried out where the amount of immunoreactive IR cells (IR) was distributed in the following scores: SCORE 0: Null immunostaining pattern was a total absence of IR cells; SCORE 1: Low pattern of immunostaining (up to 5 IR cells per field); SCORE 2: Moderate pattern of immunostaining (between 6 and 12 IR cells per field); SCORE 3: high standard of immunostaining (more than 12 IR cells per field).

-Statistical Analysis

For statistical analysis of the data, the Bioestat 5.0 program (Instituto Mamirauá, Manaus, Brazil) was used. Shapiro-Wilk test was performed to verify homoscedasticity and whether the results were parametric. After confirming the normal distribution, Student’s t statistical test was used for each evaluated value of PB, PS, PBGM. For OCN and TRAP were analyzed by Kruskal-Wallis and Student-Newman-Keuls post-test. The significance level adopted was 5% (*p* < 0.05).

## Results

-Qualitative histological evaluation of the samples 

In samples from the surgical site in the C and PBM groups, the presence of bone was observed, which was composed of bone trabeculae that were thinner in the control and quite thick in the PBM group. Interspersed between the bone trabeculae, the presence of soft tissue and biomaterial remnants was observed in both groups. In the control, there was a greater amount of soft tissue compared to the PBM group. The soft tissue present in such samples was predominantly composed of connective and adipose tissue. The remnants of the biomaterial of inflammatory cells were contacted. The photomicrographs showing the histological aspect of the samples from both groups are shown in Fig. 3.

-Histomorphometric analysis

-Percentage of bone tissue (PB).

The PBM group had a higher PB (55.3 ± 3.8) when compared to the control group (43.1 ± 5.2) (*p*<0.0001). The graph is shown in Fig. 4a.

-Percentage of soft tissue (PS).

The PBM group had a lower PS (21.8 ± 4.0) when compared to the control group (26.0 ± 2.2) (*p*<0.05). The graph is shown in Figure 4b.

 -Percentage of bone graft biomaterial (PBGM).

There was no statistically significant difference between the PBGM between the control group (22.5 ± 2.8) and the PBM group (20.8 ± 2.6) (*p*>0.05). The graph is shown in Fig. 4c.

-Immunohistochemical analysis

-Immunostaining for OCN.

The immunostaining pattern for OCN in samples from the surgical site in the different experimental groups is shown in Figure 4d. The median and interquartile deviation were: 2 (1-2), with a moderate immunostaining pattern prevailing in the control group; 3 (2-3), with a prevailing high pattern of immunostaining in the PBM group. The PBM group showed a higher pattern of immunostaining for OCN when compared to the control group (*p*<0.05).

-Immunostaining for TRAP in samples from the surgical site

The pattern of immunostaining for TRAP in samples from the surgical site in the different experimental groups is shown in Fig. 4e. The median and interquartile deviation were: 2 (1-2), with a prevailing moderate pattern of immunostaining in group control; 2 (1-2), with a moderate pattern of immunostaining prevailing in the PBM group. There was no statistically significant difference in the pattern of TRAP immunostaining between the control and PBM group (*p*>0.05).

## Discussion

The need to access the maxillary sinus, elevate the sinus membrane and fill the area of the remaining membrane-bone wall interface of the maxillary sinus with biomaterial is still discussed in the literature. A recent systematic review study with meta-analysis comparing the installation of implants with sinus lift, with or without bone filling, demonstrated a high implant survival rate in both groups, being 97.92% with sinus lift without graft and 98.73% with bone graft (26). However, sinus lift without the use of a graft showed a significantly lower gain in vertical bone height, with a mean difference of -1.73mm (*P*=0.01), and a significantly lower bone density, with a mean difference of -94.7 Hounsfield unit (*P*<0.001), compared to the side where the bone graft was used (26), which reinforces the significance of filling the maxillary sinus with the use of biomaterial.

With defined inclusion and exclusion criteria, approximately 350 previous analyzes of panoramic radiographs and computed tomography scans were performed for the selection of research participants, with 62 participants being selected. Among those selected, 54 did not participate in the survey for the following reasons: diabetes (6), tobacco (5), pandemic (13), withdrawal from continuing the study (30), and leaving 8 (eight) candidates who participated in the survey.

The present randomized controlled split-mouth clinical study demonstrated that the Cerabone® biomaterial presented excellent biocompatibility, not demonstrating an active osteoclast response and intense inflammatory reaction during the evaluation period, corroborating the findings of other studies (15,23,24,27), characterized in this study by the new bone formation observed in the control group and the presence of isolated and sparsely distributed inflammatory cells. The comparative analysis of this biomaterial with other xenogeneic biomaterials (Bio-Oss), studies demonstrated no statistically significant difference between the two biomaterials (14).

On the other hand, the PBM group showed a higher PB of about (55.3%) compared to the control group (43.1%). Furthermore, in the comparative analysis between the control groups and the PBM group, the formation of thicker bone trabeculae and reduced medullary spaces is clear, which was statistically significant and demonstrates the effective action of PBM in the area where the biomaterial was inserted. Our results confirm the effectiveness of PBM mediated by LPL in stimulating osteogenesis, also observed in other studies in animals (28,29), in humans (23,24), and in systematic review studies (30,32).

With the methodology of the present study, it is not possible to clarify the mechanisms involved in the laser-biomaterial interaction, however, some hypotheses may have contributed. Among these, the porosity of the material, the vasculature of the area, and PBM stand out. According to the manufacturer’s data, Cerabone is a product that has interconnected porosity in its granules, which facilitates hydrophilicity, adhesion, invasion, invagination, and growth of osteoprogenitor cells and vascular proliferation, promoting their total integration. Furthermore, biomaterial integration not only requires ample surface area for bone cell attachment to occur but also sufficient interparticle space for the bone to grow (33). In addition, the size of its granules with 600–900 µm allows for a greater contact area with blood and osteoprogenitor cells. Thus, it can be inferred that the LPL application protocol prior to installation and immediately after the installation of the biomaterial was effective.

Also, the laser energy applied in these two moments, both in the displaced sinus membrane and in the remaining bone wall, they were able to promote greater angiogenesis around the biomaterial. Therefore, increasing the vasculature and oxygen supply, activating osteoprogenitor cells and growth factors is possible by PBM therapy results. Since the photochemical interaction of light with the cells of the treated tissue is proven by the modulation of osteogenesis from the differentiation of mesenchymal cells of the bone marrow (19) and increasing angiogenesis, cell proliferation, osteoblastic differentiation and mineralization (21).

From the immunohistochemical point of view, there were no statistically significant differences in osteoclastic activity from TRAP-positive cells in both groups, evidencing that PBM did not interfere in bone resorption. However, PBM promoted greater ossification with a more advanced state of maturation of the bone characterized by a greater number of cells immunomarked by osteocalcin, as also reported in other studies (21,34).

The promising and relevant results observed in this study demonstrate the possibility of Cerabone® constitutes a viable alternative for the reconstruction of bone height and width of the maxillary sinus. The clinical relevance of this study suggests a greater bone volume and maturation with the use of PBM. Therefore it should be implicated in earlier oral implant rehabilitation for the patients treated with LPL application. Besides the benefits of bone formation in maxillary sinus augmentation using Cerabone®, the reduction of inflammatory signals and symptoms such as pain and oedema possibly will be extras advantages promoted by PBM (16). New research should be developed mainly in longer periods of evaluation and with the presence of installed implants.

Among the limitations of the study, the limited number of research participants stands out, which is due to the inclusion and exclusion criteria of the study and, mainly, the state of the SARS-COV2 pandemic that took place, generating insecurity for patients to accept the proposed treatment, having to come to the Institute’s clinic for care. Positively, it should be noted that there were no cases of discomfort or undesirable effects in patients treated with the biomaterial and the proposed methodology.

## Conclusions

In view of the results obtained with the methodology employed, it can be concluded that PBM mediated by LPL positively influenced bone formation in maxillary sinuses filled with the biomaterial Cerabone®, promoting greater bone formation and ossification than to the control side without the use of LPL in the 30-day evaluation period.
